# Comparative evaluation of three commercially available markerless depth sensors for close-range use in surgical simulation

**DOI:** 10.1007/s11548-023-02887-1

**Published:** 2023-05-04

**Authors:** Lukas Burger, Lalith Sharan, Roger Karl, Christina Wang, Matthias Karck, Raffaele De Simone, Ivo Wolf, Gabriele Romano, Sandy Engelhardt

**Affiliations:** 1 Department of Cardiac Surgery, Heidelberg University Hospital, Heidelberg, Germany; 2 Department of Computer Science, Mannheim University of Applied Sciences, Mannheim, Germany; 3 DZHK (German Centre for Cardiovascular Research), Heidelberg, Germany

**Keywords:** Depth sensors, Evaluation, Surgery, Surgical simulator

## Abstract

**Purpose:**

Minimally invasive surgeries have restricted surgical ports, demanding a high skill level from the surgeon. Surgical simulation potentially reduces this steep learning curve and additionally provides quantitative feedback. Markerless depth sensors show great promise for quantification, but most such sensors are not designed for accurate reconstruction of complex anatomical forms in close-range.

**Methods:**

This work compares three commercially available depth sensors, namely the Intel D405, D415, and the Stereolabs *Zed-Mini* in the range of 12–20 cm, for use in surgical simulation. Three environments are designed that closely mimic surgical simulation, comprising planar surfaces, rigid objects, and mitral valve models of silicone and realistic porcine tissue. The cameras are evaluated on *Z*-accuracy, temporal noise, fill rate, checker distance, point cloud comparisons, and visual inspection of surgical scenes, across several camera settings.

**Results:**

The Intel cameras show sub-mm accuracy in most static environments. The D415 fails in reconstructing valve models, while the Zed-Mini provides lesser temporal noise and higher fill rate. The D405 could reconstruct anatomical structures like the mitral valve leaflet and a ring prosthesis, but performs poorly for reflective surfaces like surgical tools and thin structures like sutures.

**Conclusion:**

If a high temporal resolution is needed and lower spatial resolution is acceptable, the Zed-Mini is the best choice, whereas the Intel D405 is the most suited for close-range applications. The D405 shows potential for applications like deformable registration of surfaces, but is not yet suitable for applications like real-time tool tracking or surgical skill assessment.

**Supplementary Information:**

The online version contains supplementary material available at 10.1007/s11548-023-02887-1.

## Introduction

Minimally invasive surgeries are increasingly prevalent in the recent years, as the surgical ports get smaller, enabling faster recovery times. However, maneuvering elongated surgical instruments through narrow ports demands a high skill level and dexterity from the surgeon. Surgical simulators in this regard, have the potential to reduce this steep learning curve, by enabling surgical training. Moreover, they are capable of providing quantitative feedback that further improves the surgical training process. For instance, the endoscopic video assistance used during surgical simulation can be enhanced with a 3D reconstruction of the scene. Here, RGB-D sensors, which capture color (RGB) and depth (D) images in real time, show great potential in providing such quantitative information without the need for markers or a complex setup. In particular in minimally invasive mitral valve repair (MVR), a surgery of the heart-valve, depth information facilitates surgical decision making, for example in choosing an appropriate size of ring prosthesis for the valve.

**Fig. 1:**
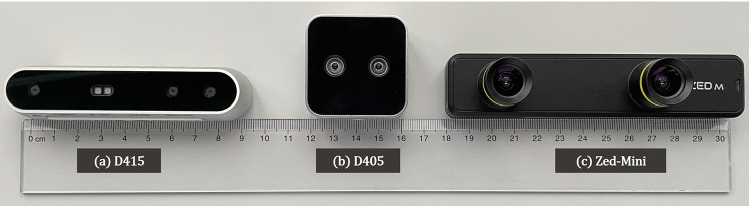
The three commercially available depth cameras that are compared in this work, namely: **a** *Intel RealSense™* D415, **b** *Intel RealSense™* D405, and the **c** *Stereolabs ZED-Mini*

**Table 1:** Selected technical specifications of the three cameras as specified by the respective manufacturers [16–18]

Specification	(a) Intel D415	(b) Intel D405	(c) Stereolabs ZED-mini
Sensor technology	Rolling shutter	Global shutter	Rolling shutter
Depth technology	Active stereoscopic	Stereoscopic	Stereoscopic
Physical dims.(mm) ($L\times D\times H$)	$99\times 20\times 23$	$42\times 42\times 23$	$124.5\times 26.5\times 30.5$
Max. frame rate	$90~\textrm{fps}$	$90~\textrm{fps}$	$100~\textrm{fps}$
Max. image resolution	$1920 \times 1080$	$1280 \times 720$	$2208 \times 1242$
Depth accuracy	$<2\%$ at 2 m	$\pm 2\%$ at 50 cm	$< 1.5\%$ up to 3 m
Ideal range	16 cm to 1000 cm	7 cm to 50 cm	10 cm to 1500 cm
FOV (H $\times$ V $\times$ D)	$69^\circ \times 42^\circ \times 77^\circ$	$87^\circ \times 58^\circ \times 92^\circ$	$90^\circ \times 60^\circ \times 100^\circ$
Manufacturer min. Z	$\sim 16$ cm	$\sim 7$ cm	$\sim 10$ cm

However, most off-the-shelf depth cameras function optimally in the range of 1 to 10 m. This hinders their adoption in surgical simulation, where typical applications like identifying fine anatomical structures, making quantitative measurements, and reconstructing dynamic scenes require accurate depth sensing in the close-range. Particularly in MVR, where surgical training is performed using patient-specific surgical simulators [1], wet surfaces and reflective materials potentially play a role in a robust 3D reconstruction of the scene.

In this work, three different commercially available depth sensors are identified that potentially work in the close-range as per manufacturer specifications, namely the *Intel RealSense™* D415, the *Intel Realsense™* D405 (Intel Corporation, Santa Clara, US), and the *Stereolabs ZED-Mini* (StereoLabs, San Francisco, US). The performance of the 3 cameras are compared for use in surgical simulation. An evaluation is performed, across three different environments containing planar surfaces (Env01), rigid objects of known geometry (Env02), patient-specific silicone mitral valve replica and porcine valves (Env03). The environments comprise static and dynamic scenes, that mimic surgical simulation in MVR. Furthermore, the influence of several parameters such as resolution, distance from the camera, and camera modes, is systematically evaluated to assess the suitability to different scenes and objects in a close-range setting.

## Related work

Multiple applications benefit from a more accurate close-range depth measurement, for example wound measurement systems [2, 3], robot-guided positioning to grip novel objects [4], or defect inspection systems [5]. Depth sensors have been used for markerless real-time tool tracking at medium range, in a multi-sensor laparoscopic training setup [6], or for automating surgical manipulation tasks [7–9] using a robotic surgical assistant, and for gesture recognition in the operating room [10]. Prior work [11–13] provides a detailed comparison of various *Intel Realsense™* cameras under different experimental setups, but however does not include the recently released D405, and is evaluated for a setting other than surgical simulation [14]. In MVR, patient-specific surgical simulators have demonstrated use in surgical training and planning [1]. Here, marker-based infrared sensors have been previously used for empirical exploration of complex 3*D* geometry [15].

## Methodology

### Depth cameras

Figure 1 shows the three different depth cameras that are compared in this work. Selected technical features of the respective cameras are provided in Table 1. The Intel RealSense™ D415 is an active stereoscopic depth camera introduced in 2018, with an infra-red emitter for active depth measurements. The camera has a rolling shutter which improves the quality of depth measurements on static scenes. The Intel RealSense™ D405 is the newest among the cameras, and is designed for applications where good accuracy and precision are important in the close-range, such as inspection and high precision picking and placing of small objects. In contrast to the D415, the D405 is only based on stereo vision and has no additional infra-red emitter. Therefore it strongly depends on good lighting conditions and well-represented texture on the objects. Both the Intel cameras, are evaluated on all possible combinations of the available post-processing filters such as *highDensity* (prioritize more depth values), *highAccuracy* (prioritise accurate depth values), etc. to identify the ones with the highest effect on the resulting depth maps. The *librealsense* Software Development Kit (SDK) version 2.51.1 is used. The Stereolabs *Zed-Mini*, similar to the D405, is a passive depth sensing tool, that is optimized for real-time depth computation. The camera provides multiple post-processing modes, such as *FILL* mode (hole filling and smoothing), *ULTRA* mode (highest depth range and better preserved *Z*-accuracy in sensing range), or *NEURAL* mode (use a neural network to improve extraction, matching and aggregation cost). The *Stereolabs* SDK version 3.7.6 is used.

**Fig. 2:**
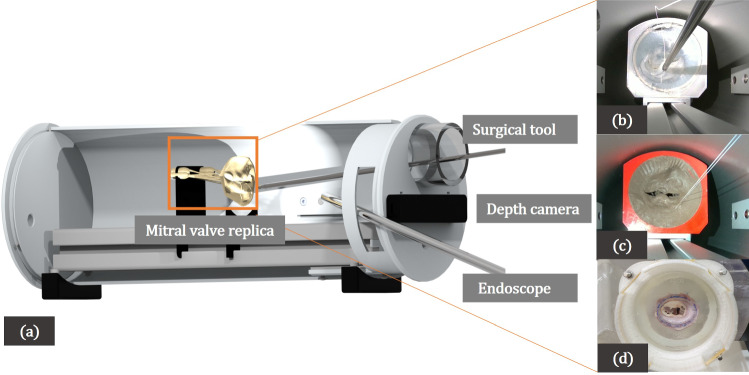
**a** The setup of the in-house mitral valve repair surgical simulator used for evaluating the depth cameras in Env03. **b** Valve01, a patient-specific translucent and **c** Valve 02, a patient-specific pigmented silicon valve replica. **d** Valve03, a mitral valve from real porcine tissue

### Evaluation criteria

This section describes the metrics used for qualitative and quantitative evaluation of the sensor performance, with respect to relevant factors such as depth accuracy, checker distance, fill rate, temporal noise, and point cloud errors.

$\textbf{Z}$*-accuracy* for a planar surface is computed by fitting a *Z*-plane to the depth values obtained, and rotating this plane to be parallel to the ground-truth plane. The *Z*-accuracy is defined as the difference or the offset between ground truth distance (GT) and the (signed) distance from a depth vertex to the fitted *Z*-plane ($D^{'}_i$) in $~\textrm{mm}$ (see Eq. 1 as per *librealsense-SDK*^1^). The best-fit *Z*-plane is computed from the Depth Quality tool^2^ of *Intel Realsense™*. A centered Region of Interest (ROI) is chosen, to focus on the planar surface (c.f. Env01, “Experimental setup” section) while cutting out the outliers from the rest of the environment. This amounts to $40\%$ of the image resolution.1*Checker distance* Another method to evaluate the accuracy of the depth computation is to measure the error in specific points of known geometry, for example with the corner points of a checkerboard, for a planar surface. For a checkerboard surface in Env01, the *Mean Absolute Error (MAE)* of the corner points is computed, between the depth measurements obtained at these corner locations ($P\!C$ in Eq. 2) and the ground-truth distance from the known geometry of the corner points (*GT* in Eqn.2). Here *N* denotes the number of points. The corner points are detected using the *findChessboardCorners* function from OpenCV, based on the Harris corner detector [19].

2 $$\begin{aligned} \text {Checker distance} = \sum _{n=1}^{N} \frac{\mid \mathrm{{dist}}_\textrm{PC} - \mathrm{{dist}}_\textrm{GT} \mid }{n} \end{aligned}$$

*Fill Rate* The fill rate of a depth sensor is the fraction of pixels that contain valid measurements within an ROI. The fill rate is critical for tasks such as segmenting objects, or measuring object dimensions. A pixel measurement is considered to be valid if it has a non-zero value, and is within $2~\textrm{cm}$ from the ground-truth distance.

*Temporal noise* helps quantify the stability of scenes over a sequence of frames, in particular for static scenes. Here, instabilities are usually noticeable on the depth-edges of the objects, specular surfaces, and motion in the scene. For our experiments, the temporal noise of the depth values is computed from static scenes of the experiments, with a quadratic difference over 10 frames.

*Point cloud comparison* To evaluate the spatial accuracy of the reconstructed anatomical structures, the point cloud obtained from the depth sensor is compared with the corresponding segmented model (see “Experimental setup” section for more details about the segmented model). Firstly, the point clouds are registered using the *CloudCompare* library using an iterative closest point (ICP) algorithm for 3D-point sets [20]. The Cloud-to-Cloud (*C*2*C*) distance is computed, defined as the euclidean distance between a point obtained from the depth sensor and the nearest point in the reference cloud. Additionally, the Cloud-to-Mesh (*C*2*M*) distance is also determined, which is the euclidean distance between each point in the obtained cloud to the nearest triangle in the reference mesh. Since the objects have a centered circular ROI (see Fig. 2b, c), the *Hough Circle Transform* from the *OpenCV* library is used to crop the measured object and additionally remove the outliers by manual inspection.

## Experimental setup

**Fig. 3:**
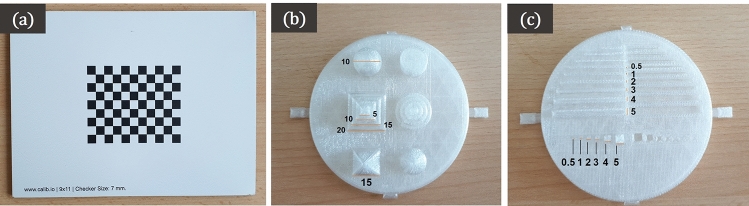
**a** Planar checker surface evaluated in Env01, with a square size of $7~\textrm{mm}$. **b** Obj01 with pyramids with squares of different depths and additionally a sphere and a cylinder of known geometry and **c** Obj02 with different gradations of cubes and rectangles, evaluated in Env02 (all dimensions annotated in mm)

*Planar surfaces (Env*01) Firstly, planar surfaces are placed in front of the camera, and the stability and the temporal errors in faithfully capturing the surface are evaluated. A flat planar surface and a checkerboard surface (see Fig. 3a) are used for this evaluation, at distances of 12, 16, 18 and $20~\textrm{cm}$ from the camera. The *Z*-accuracy, temporal noise, and fill rate for both surface types are evaluated. Additionally, for the checkerboard pattern, the checker distance is computed, as described in “Evaluation criteria” section. All the recordings were made under similar physical conditions in a room without windows, under the same room-lighting, to minimize the variations caused by external lighting conditions. Furthermore, the experiments are performed with several depth settings (*highDensity*, *highAccuracy*, and disparity shift settings for the *Intel* cameras; *NEURAL*, *PERFORMANCE*, and *ULTRA* depth modes for the *Zed-Mini*).

**Fig. 4:**
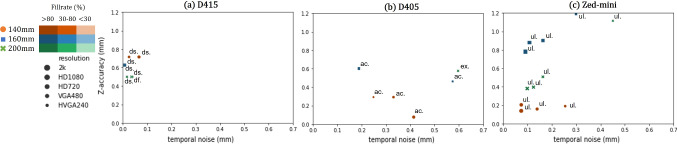
*Z*-accuracy (lower is better) versus temporal noise (lower is better) for the flat planar surface for each distance, resolution and camera model. The D405 provides the best *Z*-accuracy at a distance of $20~\textrm{cm}$ but with higher temporal noise. The D415 provides a low temporal noise but with a worse *Z*-accuracy. The *Zed-Mini* has the best fill rate among the cameras. *Modes* *pr*: *PERFORMANCE*, *df*: *DEFAULT*, *ex*: exposure, *dn*: highDensity, *ac*: highAccuracy, *ul*: *ULTRA* mode

*Rigid objects of known geometry (Env*02) In this environment, two 3D printed objects are used, namely Obj01 and Obj02 (c.f. Fig. 3b and c respectively). Obj01 contains a pattern with pyramids, cones, and spheres that mimic small objects, with a step-size of $2.5~\textrm{mm}$ ranging from 0 to $1~\textrm{cm}$. Obj02 consists of cubes and rectangles in different sizes ranging from 0.25 to $1~\textrm{cm}$. The computer-aided design (CAD) models of both Obj01 and Obj02 are made publicly available.^3^ For both the rigid objects, the *C*2*C* distance and the *C*2*M* distance are computed, as described in “Evaluation criteria”.

*Valve models (Env*03) In addition to rigid objects, an environment with different mitral valve models is additionally used for evaluation. Firstly in Valve01, a patient-specific translucent silicone replica [1] (see Fig. 2b) of the mitral valve is used. Secondly, for Valve02, a pigmented version of the silicone valve replica (see Fig. 2c) is used, to negate the reflection on a translucent valve surface leading to faulty depth measurements. The reflection could be due to different light sources such as the endoscopic light or the light from the depth camera itself. Both Valve 01 and 02 exhibit tissue-like haptic properties [1], and are segmented from the pre-operative 3D trans-esophageal echocardiogram (TEE) captured in mitral valve repair procedures. For a detailed description of how these valves are produced, the reader is referred to [1]. This environment directly mimics the surgical setting and helps evaluate the camera’s usefulness in identifying complex anatomical structures, in the presence of surgical tools and sutures in the scene (see Fig. 2). The experiments for Valve01 and Valve02 were performed under endoscopic lighting in the surgical simulator set to 70% illumination. Thirdly in Valve03, we use a porcine mitral valve to test on more realistic tissue surfaces and valvular textures (see Fig. 2d). Here, the experiments were performed with room lighting without an endoscope, as described in Env01 and Env02.

In this environment, the obtained depth measurements are evaluated across four different relevant scenes from the MVR procedure: (a) Valve inspection: In this scene for Valves 01 and 02, firstly a closed mitral valve surface is captured, and secondly an open mitral valve that is inspected with a tool (see Fig. 2a). In Valve02, a sizing tool is additionally inserted to obtain a clearer view of finer anatomical structures below the leaflet surface like the *papillary muscles* and the *chordae tendinae* (see Fig. 8a). Here, the quality of reconstruction of these fine structures is evaluated. (b) Surgical tools and needle: Different surgical tools and a fixed needle are held at different distances and angles in front of the camera and the quality of the obtained reconstruction is inspected. (c) Surgical sutures: Suturing is performed on the valve annulus i.e. around the rim of the valve, resulting in two sutures (EH7713LG Ethibond Excel Polyester Suture, Ethicon, Ohio, US) protruding out of the valve surface (see Fig. 2c). Here, it is evaluated if the depth information can be used to compute the distance between the entry and exit points of the sutures. This is an important step in tasks like automatic surgical skill assessment. (d) Ring prosthesis: Mimicking a typical step in mitral valve repair, a ring prosthesis is implanted for Valves 01 and 02, around the valve annulus (see Fig. 11a). Here, it is assessed if the depth computation reflects the placement of the ring, and if the differences in the ring and valvular surfaces can be identified with the measured depth information. For each of the scenes, the anatomical structures, outliers, and the surface shape are visually inspected. Furthermore, the depth values at the edges surrounding objects of interest such as surgical tools, sutures, and the valve annulus are also assessed. An image illustrating all the scenes used in this environment can be found in Appendix 3 of the supplementary information.

**Fig. 5:**
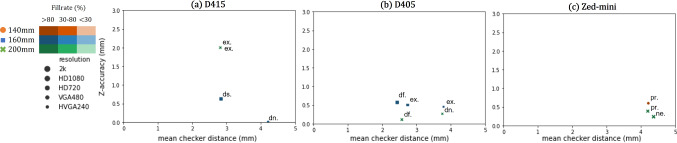
*Z*-accuracy (lower is better) versus Checker distance (lower is better) for the checkerboard planar surface for each distance, resolution and camera model. The D405 performs the best with respect to the checker distance, whereas the D415 (active stereo camera) provided the best *Z*-accuracy. The modes are denoted as, *pr*: *PERFORMANCE*, *df*: *DEFAULT*, *ex*: exposure, *dn*: highDensity, *ac*: highAccuracy, *ul*: *ULTRA* mode

**Fig. 6:**
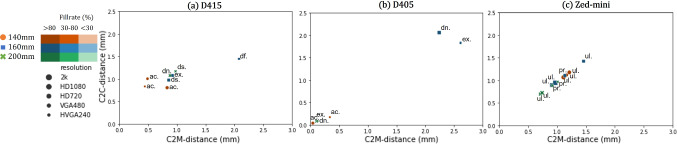
*C*2*C*-distance (lower is better) vs. *C*2*M*-distance (lower is better) for each distance, resolution and camera model. The D405 performs the best for $14~\textrm{cm}$ and $20 ~\textrm{cm}$, whereas the D415 and *ZED-mini* perform similarly. The modes are denoted as, *pr*: *PERFORMANCE*, *df*: *DEFAULT*, *ex*: *exposure*, *dn*: *highDensity*, *ac*: *highAccuracy*, *ul*: *ULTRA* mode

**Fig. 7:**
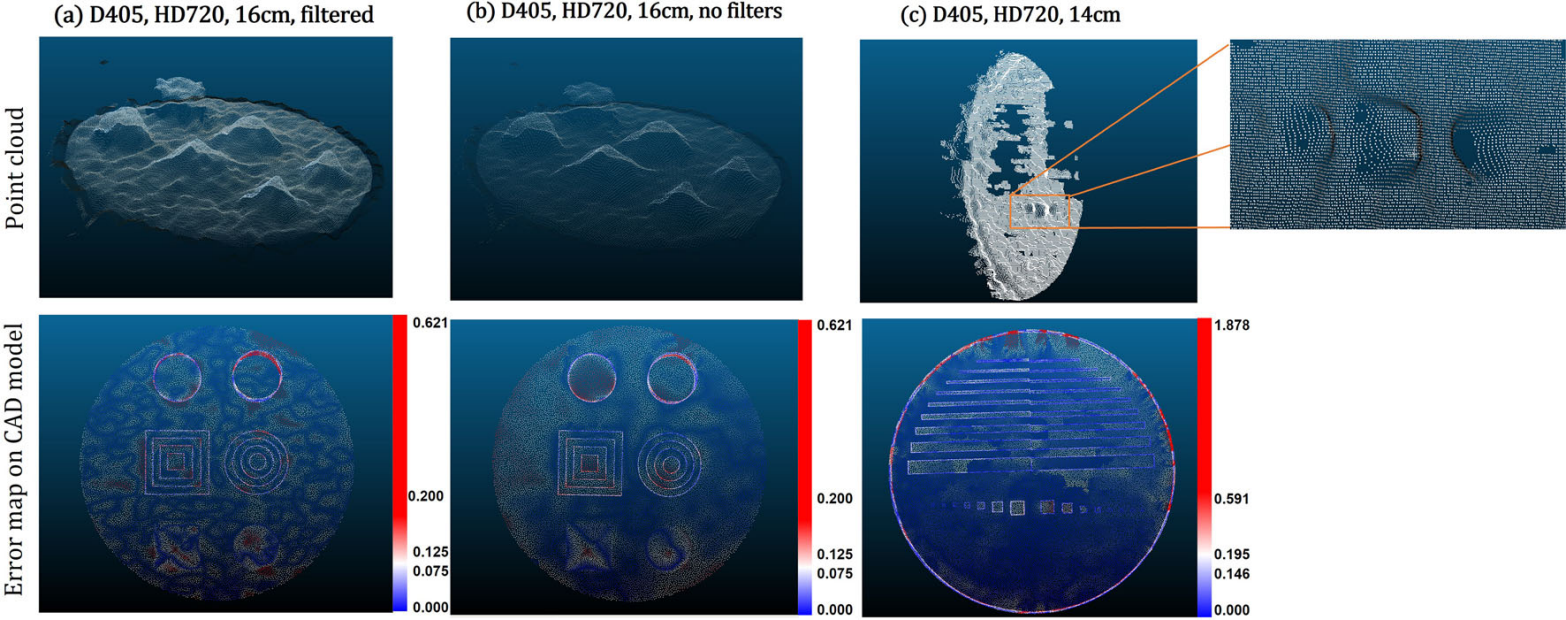
**a** and **b** show Obj01 from Env02: The point-clouds obtained with different settings and their respective error maps (in $\textrm{mm}$) while registering the respective CAD models. The conic and pyramidal structures are reconstructed roughly, but the step edges of the geometries are not resolved. Similarly, **c** shows an example of Obj02 where the corners of the cubes are not resolved

## Results

This section provides a summary of the results obtained with the best parameter setting for each camera. For a detailed tabulation of the obtained results for the different environments, the reader is referred to the supplementary information.

*Planar surfaces (Env*01) (a) Flat surface: All cameras provided reasonable results in capturing a flat plane placed perpendicular to the camera (c.f. Fig. 4). However, the best performance on a flat surface was achieved by the D405 at a distance of $20~\textrm{cm}$, with a *Z*-accuracy of $0.005~\textrm{mm}$, and the worst performance was by the D415, with an error of $0.714~\textrm{mm}$ at $14~\textrm{cm}$. Besides, the *Zed-Mini* produces a huge error with the *NEURAL*-depth mode and is otherwise able to provide similar performance to the D415. (b) Checkered surface: The D405 performed the best with a mean checker-distance error of $3.485\pm 1.575 \textrm{mm}$ with an average of 36.76 measured distances, followed by the D415 with an error of $3.644\pm 1.692 \textrm{mm}$ with 141.98 and the *Zed-Mini* with an error of $22.293\pm 30.979 \textrm{mm}$ with an average of 142 measured distances (c.f. Fig. 5). In summary, while the D405 performs the best in computing 3D distances, only a few distances were computed, possibly due to the matching algorithm unable to find enough correspondences on the checkerboard. The active stereoscopic D415, however, is able to provide a mm-level accuracy with a high fill rate. The *Zed-Mini* performs the best for the checkerboard surface, with respect to the fill rate and *Z*-accuracy in *PERFORMANCE*-mode, and the worst with the *NEURAL*-mode.

**Table 2:** A summary of the best results for each parameter and the corresponding settings under which they were observed

Parameter (mm)	Value	Camera	Distance (mm)	Resolution	Fill rate (%)	Setting
*Z*-accuracy	0.005	D405	200	HVGA	99.850	HighDensity
Checker distance	0.016	D405	160	VGA	4.862	HighAcc
Temporal noise	0.022	D415	160	HD720	99.988	Disparityshift: 292
c2c-distance	0.036	D405	140	HD720	99.850	HighAcc
c2 m-distance	0.039	D405	140	HVGA	99.850	HighAcc

**Fig. 8:**
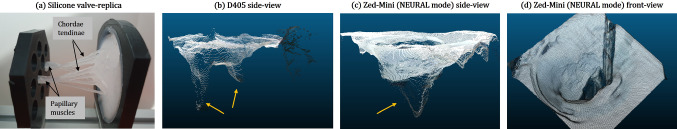
Reconstruction of a scene with Valve01, an open translucent silicone valve-replica containing the *papillary muscles* and *chordae-tendinae* as shown in (**a**). The (**b**) D405 and the (**c**) Zed-Mini are only able to sparsely reconstruct the structures, whereas the (**d**) leaflet anatomy is reconstructed well

**Fig. 9:**
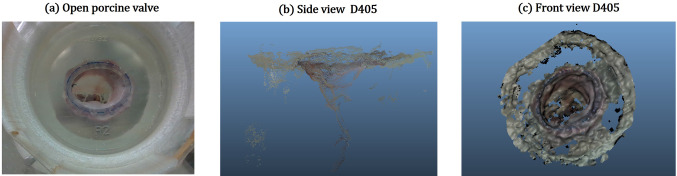
**a** A view of Valve03, an open porcine mitral valve used for evaluation in Env03, **b** side and **c** front views of the surface reconstruction obtained from the D405 at *HD*720 resolution

**Fig. 10:**
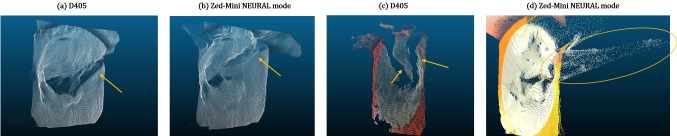
The reconstructions from D405 and the Zed-mini for a scene with Valve01 (**a** and **b**) and Valve 02 (**c** and **d**), from Env03, with the presence of surgical tools. The metallic tools are not reconstructed by any of the two cameras. The reconstruction contains holes, false or invalid depth values where the tools are supposed to be located (annotated)

**Fig. 11:**
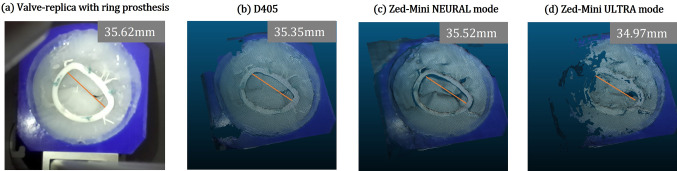
Reconstruction of Valve01, Env03 with **a** the ring prosthesis implanted on to the annulus. **b** shows a reconstruction from D405 with default settings at HD720 resolution. The *NEURAL* mode **c** is unstable over time, whereas in contrast the *ULTRA* **d** mode has a lower accuracy but is more stable over time. A comparison is shown of the measured transverse diameter (in mm) of the ring prosthesis

*Rigid objects of known geometry (Env*02) Fig. 6 shows a comparison between the *C*2*C* and *C*2*M* distance for Obj01. Here, the D405 (c.f. Fig. 6b) showed the least *C*2*C* error, at distances of $14~\textrm{cm}$ and $20~\textrm{cm}$ from the camera, with a value of $0.17~\textrm{mm}$ using the default filters setting. Although the different steps of the object surface can be reconstructed, the use of smoothing filters leads to less accurate depth values. The 4 steps of Object01 (down to $5~\textrm{mm}$, c.f. Fig. 3b) were roughly reconstructed, but the step edges were not resolved. The D415 showed a high *C*2*C* error of $5.7~\textrm{mm}$ and a very sparse depth representation of the geometric forms. For Obj02, the camera is able to resolve the depth from the first three protrusions (down to $3~\textrm{mm}$, c.f. Fig. 3c), with a *C*2*C* error of $0.15~\textrm{mm}$. Figure 7 illustrates the reconstruction results obtained for the different gradations in both objects.

A tabular summary of the best performing value for each chosen evaluation metric, and the respective camera settings under which they were observed is presented in Table 2.

*Valve models (Env*03) In Env03, unlike the previous environments, the depth information is captured while deforming the scene with surgical tools and objects, which makes quantitative evaluation challenging. We therefore qualitatively evaluate the scenes, as described in “Experimental setup”. Moreover, as a poor performance was observed from the D415 in this scenario, due to huge holes and an insufficient spatial resolution, the data from the D415 is omitted. (a) Valve inspection: Both the D405, and the *Zed-Mini* are able to reconstruct structures below the leaflet surface like the *papillary muscles* (see Fig. 8) from static scenes. However, the reconstruction is sparse, and the structures of the *chordae-tendinae* are not clearly visible from the reconstructed surface (c.f. Fig. 8 (b) and (c)). There was no noticeable difference in the reconstruction of the pigmented Valve02 surface, compared to Valve01. Moreover, Valve03 which has realistic tissue surfaces from a porcine valve, was also reconstructed by both the cameras with a few missing or inaccurate depth values (see Fig. 9). The depth of the papillary muscles were visible as shown in Fig. 9b. It is to be noted that the *NEURAL* mode of the *Zed-Mini* shows high inaccuracies in reconstructing the papillary muscles. (b) Surgical tools and needle: Both cameras are unable to reconstruct the surgical tools or the needle (see Fig. 10) for all the valves. Either 0 (invalid) or wrong depth values are observed at these locations in the scene, i.e. values that are same as the depth values of the leaflet surface. Furthermore, unsurprisingly, the depth values get noisier in locations close to the instruments, due to reflective surfaces that hinder accurate depth computation. The tip of the tools were reconstructed in the case of Valve03, but with inaccuracies near the tool shaft. (c) Surgical sutures: The sutures placed on the valve surface are not visible in the reconstructed point clouds obtained from both the cameras. In most cases, the needle and knot were not visible, and the depth values at these locations are the same as that of the mitral valve surface. The depth values near the sutures are visibly disturbed. (d) Ring prosthesis: Both the D405 and the *Zed-Mini* are able to reconstruct the valve annulus with the implanted ring prosthesis (see Fig. 11). The *NEURAL* mode of the *Zed-Mini* is unstable resulting in huge temporal outliers (see Fig. 11c). In contrast, the *ULTRA* mode (see Fig. 11 (d)) is more stable, although it reconstructs the depth with fewer accurate depth values. However, for all the cameras and the camera settings, the knots made on the sutures could not be reconstructed.

## Discussion

From the wide range of experiments performed, it can be observed that in a close-range setting, the experimental conditions and camera parameters impact the quality of depth measurements obtained. The D415 is the only active stereo camera, equipped with a laser emitter. This advantage is evident in the better performance for planar surfaces in Env01. However, it fails to accurately compute dense depth information for more complex scenes in Env02 and Env03. The camera struggles with capturing depth at close-range environments and works optimally at a distance of $20~\textrm{cm}$. For closer distances, the camera requires specific adjustments to obtain satisfactory depth measurements.

In comparison to the D405, the *Zed-Mini* is less accurate but always shows a higher fill rate than the other two cameras. Besides, it provides an easy switch between different depth modes, which have different advantages. For example the *ULTRA*-mode provides measurements with near sub-millimeter accuracy, but with sparse depth information. The newly released *NEURAL*-mode (*ZED*-SDK version 3.6) can reproduce a good spatial resolution with near $100\%$ fill-rate for larger surfaces like that of the mitral valve leaflet. However, the performance of this mode works best at the center of the scene, and fails near the edges with high error. This could be observed in Obj01, and Obj02 of Env02 in “Results” section.

The latest of the cameras, the D405, is also the one with the closest minimum working distance as per manufacture specification ($7~\textrm{cm}$, c.f. Table 1). This is reflected in the performance in the different environments in comparison to the other cameras, even though reconstructing complex scenes was sub-par when surgical tools obstruct the camera view. Furthermore, it can be observed that the post-processing filter of the D405, while able to remove temporal noise, introduces strong smoothing effects which interfere with an accurate depth reconstruction, especially on the edges.

In contrast to surgical simulation, the use of the depth sensors in an intra-operative setting is limited by different challenges. Firstly, adequate sterile contraptions are required to circumvent safety concerns. Secondly, logistic and size limitations exist in maneuvering the camera intra-operatively with narrow surgical ports.

## Conclusion

This work evaluates the performance of three commercially available depth sensors for close-range use in surgical simulation, namely the *Intel RealSense™* D415, *Intel Realsense™* D405, and the *Stereolabs ZED-Mini*. The *Zed-Mini* is recommended when working with a lower spatial resolution. In particular, the *NEURAL* mode is a good compromise between the *Z*-accuracy and fill rate, but fails with huge outliers in case of flat surfaces or small objects. The D405 is the most-suited for close-range use in settings that mimic minimally invasive mitral valve repair simulation. The D405 is able to reconstruct anatomical structures such as the valve leaflet and the ring prosthesis, which shows potential for applications like real-time deformable registration. The reconstruction fails, however, in case of reflective surfaces such as surgical tools and thin structures like the sutures. This makes it difficult to use for applications such as depth-based real-time tool tracking in the close-range, or for surgical skill assessment.

The future work involves further validating the best performing depth sensors under real-time conditions. Besides, emerging depth sensing techniques such as light field cameras are robust to reflections and occlusions, and show promising directions for close-range use in surgical simulation. Future improvements in the form factor of these cameras could provide impetus to close-range intra-operative applications.

## Supplementary Information

Below is the link to the electronic supplementary material.

Supplementary file 1 (pdf 2198 KB)

## Footnotes

1 https://github.com/IntelRealSense/librealsense.

2 https://dev.intelrealsense.com/docs/camera-depth-testing-methodology.

3 https://github.com/Cardio-AI/depth-sensor-evaluation.
