# Supplementary material for: Comparative evaluation of three commercially available markerless depth sensors for close-range use in surgical simulation
Source: Int J Comput Assist Radiol Surg. 2023 May 4;18(6):1109–18. doi: 10.1007/s11548-023-02887-1 (PMC10284995; doi:10.1007/s11548-023-02887-1)
Supplement: Supplementary file 1 — (pdf 2198 KB) [file 11548_2023_2887_MOESM1_ESM.pdf]

Supplementary Information

Comparative evaluation of three commercially available markerless depth sensors for close-range use in surgical simulation

International Journal of Computer Assisted Radiology and Surgery

Lukas Burger[1,2†], Lalith Sharan[1,3*†], Roger Karl[1,3], Christina Wang[1], Matthias Karck[1], Raffaele De Simone[1], Ivo Wolf[2], Gabriele Romano[1] and Sandy Engelhardt[1,3]

[1]Department of Cardiac Surgery, Heidelberg University Hospital, Heidelberg, Germany.
[2]Department of Computer Science, Mannheim University of Applied Sciences, Mannheim, Germany.
[3]DZHK (German Centre for Cardiovascular Research), partner site Heidelberg/Mannheim, Germany.

Corresponding author(s). E-mail(s): lalithnag.sharangururaj@med.uni-heidelberg.de;
†These authors contributed equally to this work.

Appendix A Results

A.1 Planar surfaces (Env 01)

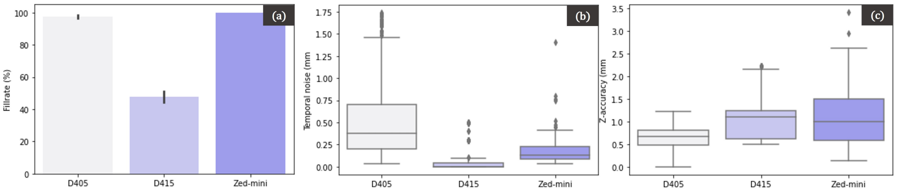

Fig. A1 Mean metrics computed over all camera modes and settings (a) Fill-rate (b) Temporal noise (c) Z-accuracy. The *NEURAL* mode of the *Zed-Mini* is omitted in the mean computation for all the metrics, as it leads to huge outliers, thereby making it difficult to view the other distributions

Fig. A1 shows the mean performance over all camera settings, and all distances for the 3 depth sensors, based on the fill rate (Fig. A1 (a)), temporal noise (Fig. A1 (b)), and Z-accuracy (Fig. A1 (c)).

Fill rate: The *ZED-Mini* performed the best, achieving a 100% fill rate with all configurations. This is followed by the $D405$, with a fill rate of about 80% in the *high accuracy* mode. The $D415$ however, was only able to achieve a fill rate above 80% when the disparity shift settings were adjusted, and otherwise provided a fill rate of $< 40\%$ (see Fig.A1 (a)).

Temporal noise: From Fig. A1 (b), we see that for every distance from the camera, the $D415$ is able to provide the least temporal noise of 0.022mm at a distance of 16cm and a resolution of $HD720$, with the *disparityshift* setting at 292. This is also reflected in the overall performance, with a mean error of 0.0446 ± 0.101mm. This is followed by the *ZED-Mini* (0.1856 ± 0.179mm), and the $D405$ (0.5065 ± 0.4082mm) (see Fig. A1 (b)).

Z-accuracy: The $D405$ achieved best performance with an accuracy of 0.005mm at an optimal range of 20cm in $HVGA$ resolution with the *high-Density* setting, and a mean accuracy of 0.637 ± 0.259mm (see Fig.A1 (c)), followed by the $D415$ (1.073 ± 0.439mm for the valid depth data), and finally the *Zed-Mini* (8.426 ± 20.759mm). Here, the *NEURAL* depth-mode leads to huge inaccuracies towards the edges of the target, causing multiple outliers, and is therefore omitted from the mean computation shown in Fig. A1 (c). However, without this mode the *Zed-Mini* achieves a similar mean error to the $D415$ (1.132 ± 0.661mm).

Table A1 Best results for the flat planar surface from Env01, for each distance and all the cameras with respect to Z-accuracy.

Name	Distance (mm)	Resolution	Setting	Fill rate (%)	Z-accuracy (mm)	Temporal noise (mm)
D405	120	HD720	highAcc	100.000	0.075	0.414
D405	140	HVGA	highAcc	99.910	0.463	0.574
D405	200	HVGA	highDensity	99.850	0.005	1.435
D415	140	HD720	disparityshift: 338	99.982	0.714	0.066
D415	160	HD720	disparityshift: 292	99.988	0.625	0.022
D415	200	HD720	disparityshift: 124	99.962	0.500	0.076
Zed-mini	140	HD2K	Depth mode: *ULTRA*	100.000	0.130	0.074
Zed-mini	160	HD720	Depth mode: *NEURAL*	100.000	0.580	0.178
Zed-mini	200	VGA	Depth mode: *NEURAL*	100.000	0.157	0.472

Table A2 Best results for the checkerboard surface from Env01, for each distance and all the cameras with respect to checker distance.

Name	Distance (mm)	Resolution	Setting	Fill rate (%)	Z-accuracy (mm)	Checker distance (mm)
D405	160	VGA	highAcc	4.862	0.381	0.016
D405	180	VGA	highDensity	0.031	6.160	0.021
D405	200	HVGA	highDensity	8.073	0.085	0.036
D415	160	VGA	disparityshift: 134	99.980	0.100	1.239
D415	200	VGA	disparityshift: 82	99.994	0.500	1.214
Zed-mini	160	HD720	Depth mode: *NEURAL*	100.000	1.117	2.480
Zed-mini	180	HD2K	Depth mode: *PERFORMANCE*	100.000	0.133	1.000
Zed-mini	200	HD1080	Depth mode: *PERFORMANCE*	100.000	0.386	1.117

A.2 Rigid objects of known geometry (Env 02)

Table A3 Best results for each distance and all cameras with respect to the $C2C$ distance.

Name	Distance (mm)	Resolution	Setting	Fill rate (%)	c2c-distance (mm)
D405	140	VGA	highAcc	29.959	0.036
D405	160	HVGA240	exposure	94.779	1.830
D405	200	HD720	highAcc	39.597	0.077
D415	140	HD720	disparityshift: 124	32.636	0.805
D415	160	HD720	disparityshift: 134	72.392	0.975
D415	200	VGA	disparityshift: 82	82.723	1.076
Zed-mini	140	2K	Depth mode: *PERFORMANCE*	100.000	1.069
Zed-mini	160	HD1080	Depth mode: *ULTRA*	100.000	0.892
Zed-mini	200	HD1080	Depth mode: *ULTRA*	100.000	0.696

Table A4 Best results for each distance and all cameras with respect to the $C2M$ distance.

Name	Distance (mm)	Resolution	Setting	Fill rate (%)	c2m-distance (mm)
D405	140	VGA	highAcc	58.481	0.039
D405	160	VGA	highAcc	100.000	2.240
D405	200	HD720	highAcc	39.597	0.106
D415	140	HVGA240	highAcc	42.609	0.444
D415	160	HD720	disparityshift: 134	72.392	0.859
D415	200	VGA	disparityshift: 82	82.723	0.882
Zed-mini	140	2K	Depth mode: *PERFORMANCE*	100.000	1.112
Zed-mini	160	HD1080	Depth mode: *ULTRA*	100.000	0.910
Zed-mini	200	HD1080	Depth mode: *ULTRA*	100.000	0.713

A.3 Valve Models (Env 03)

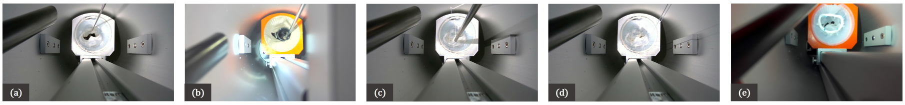

Fig. A2 The surgical scenes used for evaluation of a static surgical simulator with patient-specific silicone valve replica of the mitral valve for mitral valve repair. (a) Surgical tools (b) Open mitral valve with spacer (c) Stitching procedure (d) Sutures in the scene (e) Ring prosthesis

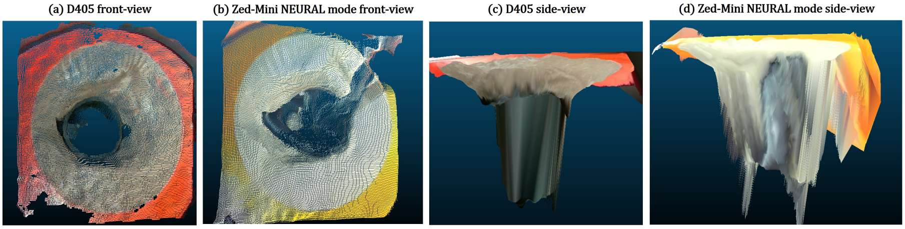

Fig. A3 Reconstruction of a scene with the pigmented silicone valve-replica (Valve02), where a sizing object is inserted into the valve to open up the view of the chordae. The object is more recognizable from the *D*405 ((a) and (c)), whereas the *NEURAL* mode from the *Zed-Mini* provides a patchy reconstruction ((b) and (d))

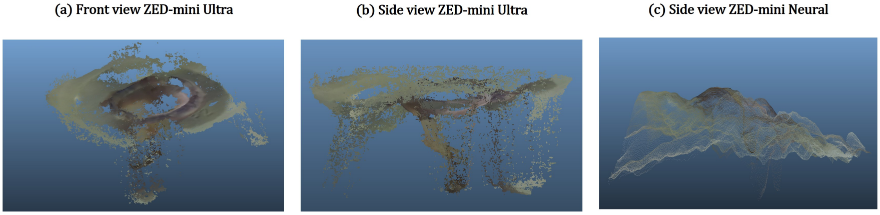

Fig. A4 Depth measurements from the **Zed-mini** of a porcine mitral valve (Valve03) in HD720 resolution. (a)Front view of the point-cloud with *Ultra*-mode (b) Side view of the point-cloud with *Ultra*-mode (c) Side view of the point-cloud with *Neural*-mode
